# PETN-Induced Antioxidative Properties in Endothelial Cells as a Target for Secondary Prevention of Endothelial Dysfunction in Pregnancy

**DOI:** 10.3389/fphys.2022.882544

**Published:** 2022-05-30

**Authors:** Veronika Teichert, Silke Große, Anna Multhaup, Jasmin Müller, Ruby N. Gutierrez-Samudio, Diana M. Morales-Prieto, Tanja Groten

**Affiliations:** ^1^ Placenta Lab, Department of Obstetrics, University Hospital Jena, Jena, Germany; ^2^ Department of Dermatology, University Hospital Jena, Jena, Germany

**Keywords:** NO-donor, pentaerytrithyltetranitrate, heme oxygenase-1, fetal growth restriction, preeclampsia

## Abstract

The NO-donor Pentaerytrithyltetranitrate (PETN) has vasodilatative properties and direct protective effects on endothelial cells. We formerly demonstrated that PETN, given to pregnant women during the second and third trimester, influences endothelial dysfunction related pregnancy complications like preeclampsia (PE) and fetal growth restriction (FGR). PETN treatment showed to delay PE to late pregnancy and achieved a profound risk reduction for FGR and/or perinatal death of 40%. The aim of this study was to confirm the effect of PETN on endothelial cell dysfunction at molecular level in an experimental approach. To induce endothelial dysfunction HUVEC were treated with 10 U/l of thrombin in the presence or absence of PETN. qRT-PCR analysis showed that PETN induced the expression of heme-oxygenase-1 and superoxide dismutase two but not endothelial NO-synthase under basal conditions. The induction of antioxidant proteins did not change basal reactive oxygen species (ROS) levels as measured by MitoSOX™ staining. PETN treatment significantly delayed the thrombin-induced disruption of the endothelial monolayer, determined using the xCELLigence^®^ and attenuated the disrupting effect of thrombin on tubular junctions as seen in a tube-forming assay on Matrigel™. In western-blot-analysis we could show that PETN significantly reduced thrombin-induced extracellular signal-regulated kinase activation which correlates with reduction of thrombin-induced ROS. These experimental results establish the concept of how PETN treatment could stabilize endothelial resistance and angiogenic properties in pregnancy-induced stress. Thus, our results underscore the assumption, that the shown clinical effects of PETN are associated to its endothelial cell protection.

## Introduction

Preeclampsia (PE) and fetal growth restriction (FGR) still remain to be main causes of maternal and fetal morbidity and mortality associated with pregnancy, labor and birth. Endothelial dysfunction is recognized to be the primary cause of these placenta associated pregnancy diseases (Ahmed and Ramma, 2015). The imbalance of endothelial protective and antiangiogenic factors in the mother’s circulation leads to peripheral vasoconstriction and subsequent hypertension. The systemic increase in endothelial permeability results in edema and endothelial activation induces the formation of microthrombi, causing malperfusion and damage to endorgans, such as the placenta, brain, kidney and liver (Duley, 2009; Young et al., 2010; Ahmed and Ramma, 2015). This pathophysiological cascade results in the maternal symptoms of PE: hypertension, coagulopathy and multiorgan disorder in a previously normotensive woman (Steegers et al., 2010) and if the placenta is affected, placental malperfusion with consequent FGR.

It has been observed, that preeclamptic changes are accompanied by a reduced endothelial production of nitric oxide (NO), the main vasodilator substance released by the endothelium. Women with PE show declining NO bioavailability, while the plasma concentration of the main vasoconstrictor substance, endothelin-1, increases (Sepulveda et al., 2017). Under healthy conditions NO is produced by the endothelial NO-synthase (eNOS) and endothelial cell function is mainly dependent on correct eNOS function and activity (Daiber et al., 2019). There are several mechanisms by which NO production and NO-bioavailability are reduced. Oxidative stress—one characteristic feature of PE (Ahmed and Ramma, 2015)—is one factor which leads to eNOS uncoupling and thus reduced activity (Wenzel et al., 2007; Li et al., 2013; Daiber et al., 2017; Luo et al., 2019). Oxidative stress is caused by high levels of reactive oxygen species (ROS) that are generated as by-products of cellular metabolic or enzymatic reactions and in higher concentrations under conditions of cellular stress. One of the main sources of ROS is the enzyme NADPH-Oxidase (Bedard and Krause, 2007; Buvelot et al., 2019). A reduction of high ROS levels leads to the restoration of normal eNOS function and consequently to a normal endothelial cell function (Forstermann and Munzel, 2006; Kar et al., 2012).

The importance of oxidative stress and endothelial dysfunction in the pathophysiology of PE has prompted the development of therapeutic approaches targeted to restore the redox equilibrium like Vitamins, Proton Pump Inhibitors and Aspirin. The organic nitratester pentaerythrityltetranitrat (PETN), which has been used as angina pectoris therapy for many years, is a NO-donor, as well as an antioxidative and endothelium stabilizing drug. It enhances endothelial function through antiproliferative and antiapoptotic mechanisms (Oppermann et al., 2009); amongst others through activation of the antioxidant heme oxygenase-1 (HO-1) (Dragoni et al., 2007; Schuhmacher et al., 2010). HO-1 catalyzes the degradation of heme to ferritin, CO and bilirubin in a rate limiting step (Sikorski et al., 2004; Hull et al., 2014; Daiber and Munzel, 2015). CO acts as a vasodilator and bilirubin has additional antioxidative properties, potentiating the antioxidative effect of HO-1. Thus HO-1 induced by PETN might protect from endothelial dysfunction.

Consequently, in a clinical prospective, randomized, placebo-controlled, double-blinded pilot study at the Department of Obstetrics, Jena University Hospital, Germany we tested the effectiveness of PETN for secondary prevention of FGR, PE and preterm birth in 111 pregnant women who presented an abnormal placental perfusion at 19–24 weeks of gestation, indicating a risk for placenta associated pregnancy complications. In this study PETN showed to reduce the risk of severe FGR and perinatal death by 39%, and for preterm delivery before 32 weeks of gestation by 70% (adjusted OR 0.204; 95% CI 0.052–0.801). (Schleussner et al., 2014; Bowkalow et al., 2018). Although, the total number of patients developing PE did not differ between groups, onset of PE was delayed and severity reduced. Additionally, treatment with PETN led to an improved placental perfusion (Bowkalow et al., 2018). These data profoundly suggest that PETN is effective in the treatment of endothelial dysfunction during pregnancy by influencing endothelial function.

The aim of this experimental study was to investigate the effect of PETN on endothelial cell function *in vitro* and thus, to confirm the hypothesis of an endothelial protective effect of PETN at a molecular level.

## Materials and Methods

### Cell Culture and Treatment

Human umbilical vein endothelial cells (HUVEC) were purchased from PromoCell. The manufacturer works according to the Declaration of Helsinki “Ethical principles for Medical Research Involving Human Subjects” (1964). No patients were involved in this study. HUVEC were cultured in Endothelial Cell Growth Medium (ECGM) containing Supplement Mix (PromoCell) and 10% FCS (Sigma-Aldrich). HUVEC were incubated under standard culturing conditions (37 °C, 5% CO_2_, humidified atmosphere). HUVEC were cultured on cell culture plates (Nunclon™ Delta Surface 20,8 cm^2^) 4 × 10^5^ per plate. At 100% confluence HUVEC were pretreated with 50 µM PETN (Dotticon^®^) or DMSO (Sigma-Aldrich) for 24 h. For experiments in which the basal effect of PETN was investigated cells were then analysed. For stimulation experiments cells were then treated with 10 U/ml thrombin (Sigma-Aldrich) in serum starved medium (ECGM without Supplement Mix or FCS) for 2–30 min depending on the experiment in the presence or absence of PETN. Thrombin stimulation is acknowledged as an established model for PE *in vitro* as described by Huang et al. (2015) and others before (Zhao et al., 2012; Brunnert et al., 2021). Thrombin was used to mimic PE-induced endothelial dysfunction.

### RNA Isolation and qRT-PCR

After HUVEC were treated with PETN or DMSO for 24 h RNA was isolated by using TRIzol reagent (Invitrogen) following manufacturer’s instructions. Total RNA concentration was determined using the QIAxpert System (QIAgen). Samples with A260/A280 ratio >1.8 were stored at −80°C until processed. The expression of mRNA levels was determined by reverse transcription using High-Capacity RNA-to-cDNA™ Kit (Applied Biosystems). Quantitative real-time PCR was performed using TaqMan assays (HO-1, Assay ID: Hs01110250_m1, eNOS, Assay ID: Hs01574665_m1, superoxide dismutase 2 (SOD2) Assay ID: Hs00167309_m1 and GAPDH, Assay ID: Hs02758991_g1) and TaqMan Universal PCR Master Mix reagents (Applied Biosystems). qRT-PCR was run on a Mx3005P qPCR System (Applied Biosystems). Expressions of all mRNA levels were normalized using the 2^−ΔΔCt^ method relative to GAPDH.

### Reactive Oxygen Species Detection With MitoSox™

HUVEC were seeded in 8-chamber Culture Slides (Corning), 50 000 cells in 400 µl of ECGM containing Supplement Mix and 10% FCS per well, and left overnight. They were then incubated with 50 µM PETN or DMSO for 24 h. The basal effect of PETN was then analyzed. For investigation of the thrombin effect cells were treated with 10 U/ml thrombin in serum starved medium (ECGM without Supplement Mix or FCS) for 10 min. For ROS detection MitoSOX™ (Thermo Fisher) was added at a concentration of 5 µM in HBSS/Ca/Mg (Sigma Aldrich) and left for 10 min at 37°C protected from light. The working solution was removed, wells were washed three times with PBS, fixated with 5% formaldehyde solution (Thermo Fisher), and cells were covered using VectaShield HardSet™ Antifade Mounting Medium with DAPI (Vector). Four representative pictures were taken per slide. Following photodocumentation, images were then evaluated using the ImageJ software (National Institute of Health).

### Permeability Assay

HUVEC were seeded to E-Plates^®^-16 (xCELLigence^®^, Acea Biosciences Inc.) at a density of 10^4^ per well. Change of density of cells leads to a change of impedance, represented as cell index. To analyze a change of density after treatment normalized cell index was used. After pre-incubation in ECGM containing Supplement Mix and 10% FCS with 50 µM PETN or DMSO respectively for 24 h. To compare the formation of the barrier between control and PETN-treated cells cell index was then measured and compared. To analyze the effect of thrombin on endothelial barrier cells were treated with 10 U/ml thrombin in serum starved medium (ECGM without Supplement Mix or FCS) for up to 30 min. Barrier disruption leads to decreased cell index. The mean of the normalized cell index of duplicates was used for statistical analysis. Values were recorded over time as linear data.

### Angiogenesis Assay

Culture plates (BD Falcon^®^-12-Well non-tissue treated Cell Culture Plates, BD Biosciences) were coated with 400 µl Matrigel™ (BD Matrigel™ Basement Membrane Matrix, BD Biosciences) per well and incubated in ECGM with Supplement Mix and 10% FCS overnight. 1.6 × 10^5^ HUVEC cells, labelled with CellTracker™ Green CMFDA Dye (Thermo Fisher Scientific) were added to each well and incubated with 50 µM PETN or DMSO for 24 h to allow forming of a network of capillary-like structures. Following photodocumentation to compare the PETN effect on formation of capillary-like structures the networks were treated with 10 U/ml thrombin in serum starved medium (ECGM without Supplement Mix or FCS) and changes in network structure were analyzed at 5, 10, 20, and 30 min. Captured images were analyzed using the angiogenesis-analyzer-tool of ImageJ (National Institute of Health) quantifying the number of nodes in the network which were then normalized to time point 0.

### Western Blot

For western blot experiments HUVEC were pretreated with PETN or DMSO for 24 h followed by incubation in serum starved medium containing 50 µM PETN or DMSO for 4 h. Serum starved HUVEC were then treated with 10 U/ml thrombin. For protein analysis the reaction was stopped after 2 min by adding phosphatase inhibitor (Serva Electrophoresis) (1:100) and cell pellets were produced. For western blot analysis cell pellets were lysed in RIPA buffer (NaCl 0.2 M, sodium desoxycholate 1%, Triton X-100 1%, SDS 0.1%, Tris-HCl 0.05 M pH 7.5, EDTA 4 mM, proteinase inhibitor mix, phosphatase inhibitor) on ice for 30 min. The supernatant was used for analysis and western blot was performed as previously described (Multhaup et al., 2018). The antibodies used were directed against extracellular signal-regulated kinase (ERK) (Cell Signaling, 9102) at 1:2000, P-ERK (Cell Signaling, 4377) at 1:2000. Label application was conducted with horseradish peroxidase conjugated anti-rabbit IgG (Cell Signaling, 7074) in 5% (w/v) milk buffer at 1:10^4^. Detection was performed using the MF-ChemiBIS 3.2 detection system (biostep, DNR Bio-Imaging-Systems) and visualized *via* GelCapture Acquisition Software (DNR Bio-Imaging-Systems Version 5.1).

### Statistical Analysis

All data are from at least three independent experiments and shown as mean values ±SEM. Statistical analysis was performed using Sigma Plot 14.5 software. An unpaired t-test was performed for experiments with two conditions (control vs. PETN). For experiments with more parameters (control vs. PETN and time or thrombin-treatment) two-way repeated measurement (RM) ANOVA followed by Holm-Sidak’s multiple comparisons test was performed. *p* values <0.05 were considered statistically significant.

## Results

### Pentaerythrityltetranitrate Effects in Endothelial Cells

**FIGURE 1:**
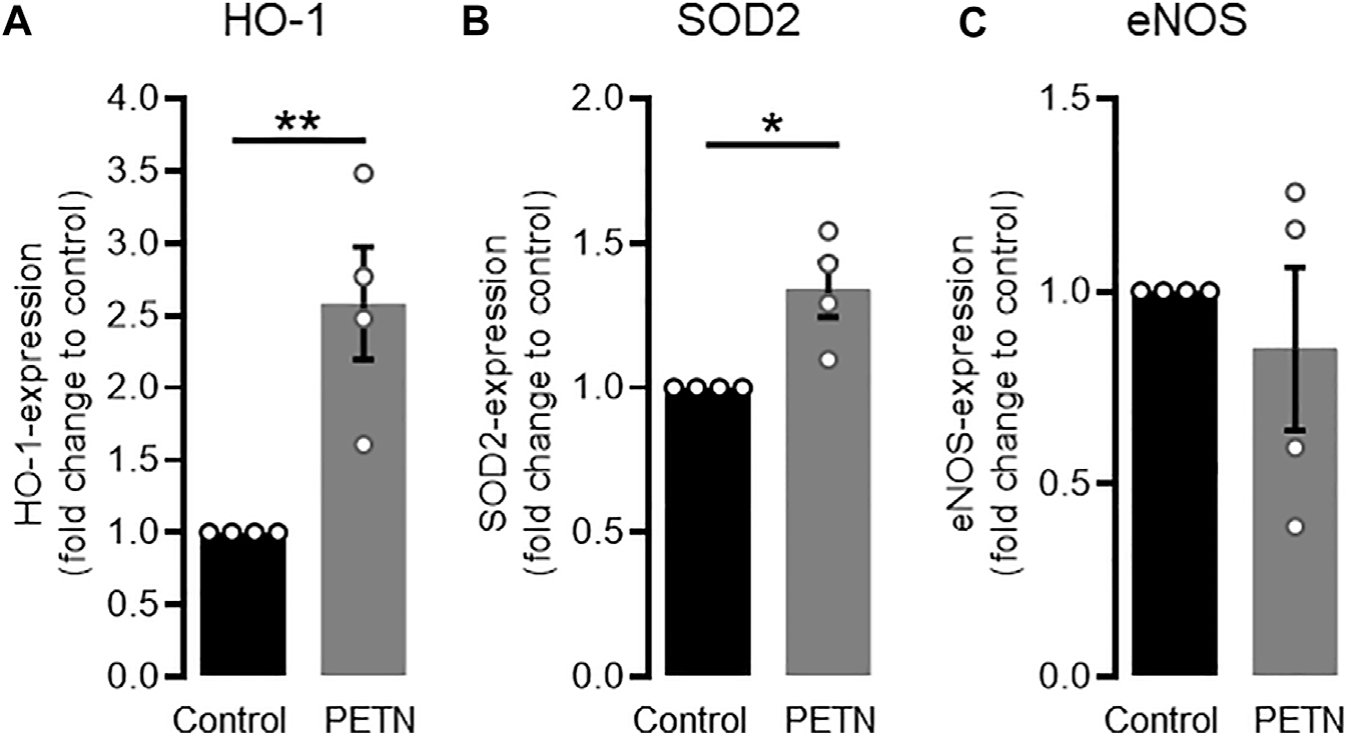
PETN treatment leads to upregulation of antioxidative proteins. RT-PCR data shows that treatment of endothelial cells with 50 µM PETN for 24 h leads to a significant 2.58 ± 0.34-fold induction of HO-1 mRNA [**(A)**, **p = 0.006] and 1.34 ± 0.08-fold induction of SOD2 mRNA expression [**(B)**, *p = 0.012]. Expression of eNOS mRNA, is unchanged **(C)** (*n* = 4, expression normalised to GAPDH and control, mean value ±SEM, unpaired t-test).

The aim of our study was to investigate if PETN has a protective effect on endothelial cells at molecular level. For this we treated HUVEC with PETN at a concentration of 50 µM for 24 h qRT-PCR revealed that this treatment led to a significant 2.58 ± 0.34-fold upregulation of HO-1 mRNA under untreated conditions (*p* = 0.006, Figure 1A). This was accompanied by a significant 1.34 ± 0.08-fold upregulation of the antioxidative SOD2 (*p* = 0.012, Figure 1B). However there was no change in the expression of eNOS mRNA (Figure 1C).

**FIGURE 2:**
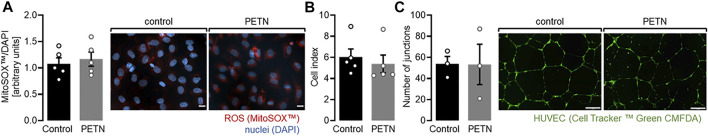
PETN treatment does not induce abnormal endothelial cell behaviour. **(A)** Treatment of HUVEC with 50 µM PETN for 24 h does not influence basal ROS production (*n* = 5, representative picture and quantification mean value ±SEM, red = MitoSOX™, blue = DAPI, scale bar = 20 µm) **(B)** Treatment of endothelial cells with 50 µM PETN for 24 h does not change the basal barrier as seen by unchanged cell index measured by the xCELLigence^®^ system (*n* = 5, cell index ±SEM). **(C)** In Matrigel™, treatment of HUVEC with 50 µM PETN for 24 h does not change the basal number of tubular junctions (*n* = 3, representative pictures, scale bar = 200 μm, and number of junctions mean value ±SEM).

The observed upregulation of the antioxidative proteins HO-1 and SOD2 led to the assumption that ROS levels might be lower in HUVEC treated with PETN. However a staining using the MitoSOX™ reagents revealed, that there was no difference in ROS levels between untreated endothelial cells and HUVEC that were treated with 50 µM PETN for 24 h (Figure 2A).

Because one major problem of PE is the systemic increase in endothelial permeability we wanted to investigate whether PETN influences endothelial barrier. We used the xCELLigence^®^ system to measure the stability of the endothelial barrier. HUVEC seeded on gold electrodes led to the formation of a stable monolayer as measured by a constant cell index. When HUVEC were seeded in medium containing 50 µM PETN and incubated for 24 h the monolayer showed a comparable cell index and no signs of an abnormal monolayer formation (Figure 2B).

In addition, an angiogenesis assay where HUVEC were embedded into Matrigel™ showed that either cells, with or without treatment of PETN formed a stable network of endothelial tubules and no difference in the number of tubular junctions could be observed. While antioxidant defense mechanisms are upregulated in HUVEC after 24 h of treatment with 50 µM PETN no abnormal behavior of the cells could be detected under basal conditions (Figure 2C).

### Pentaerythrityltetranitrate in Endothelial Cells Under Stress Conditions

After we could show that PETN did not lead to abnormal endothelial cell behavior under basal conditions we wanted to investigate if it shows protective effects under stress conditions. To mimic conditions that lead to a breakdown of endothelial barrier, as observed under preeclamptic conditions we used thrombin at a high concentration of 10 U/ml, which is known to lead to a disruption of the endothelial barrier.

**FIGURE 3:**
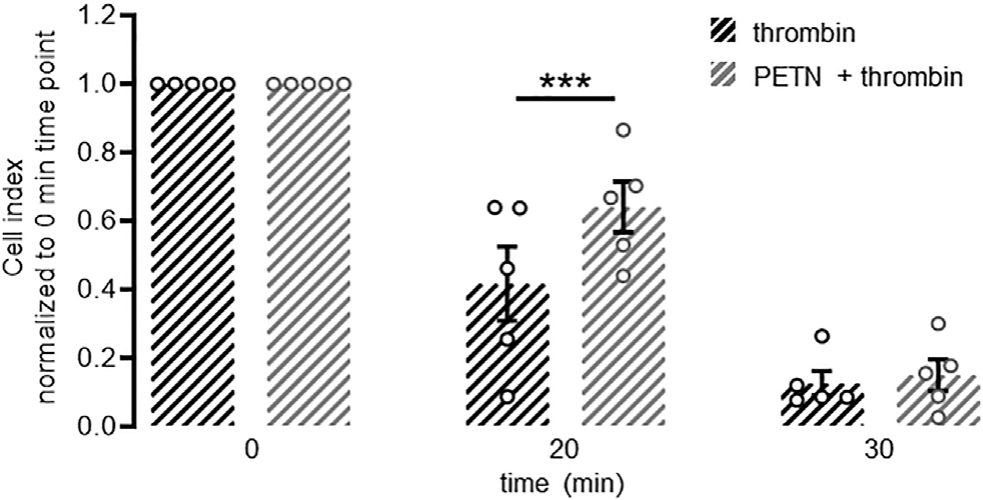
PETN treatment delays thrombin-induced barrier breakdown. Endothelial cells seeded on gold electrodes form a stable barrier as measured by cell index using the xCELLigence^®^ system. Treatment with 10 U/ml thrombin leads to a fast and almost complete disruption of this barrier as seen by a strong reduction in normalised cell index (black hatched bars). Pretreatment of endothelial cells with 50 µM PETN for 24 h (grey hatched bars) transiently but significantly protects from thrombin-induced barrier disruption (***p < 0.001 compared to thrombin). (*n* = 5, normalised cell index, mean value ±SEM, two-way RM ANOVA followed by Holm-Sidak’s multiple comparisons test).

Using the xCELLigence^®^ system we measured the breakdown of a stable endothelial monolayer 20 and 30 min after addition of 10 U/ml thrombin. When HUVEC were pretreated with 50 µM PETN for 24 h before treatment with thrombin, however, the breakdown of the endothelial barrier was significantly delayed (*p* < 0.001, Figure 3).

**FIGURE 4:**
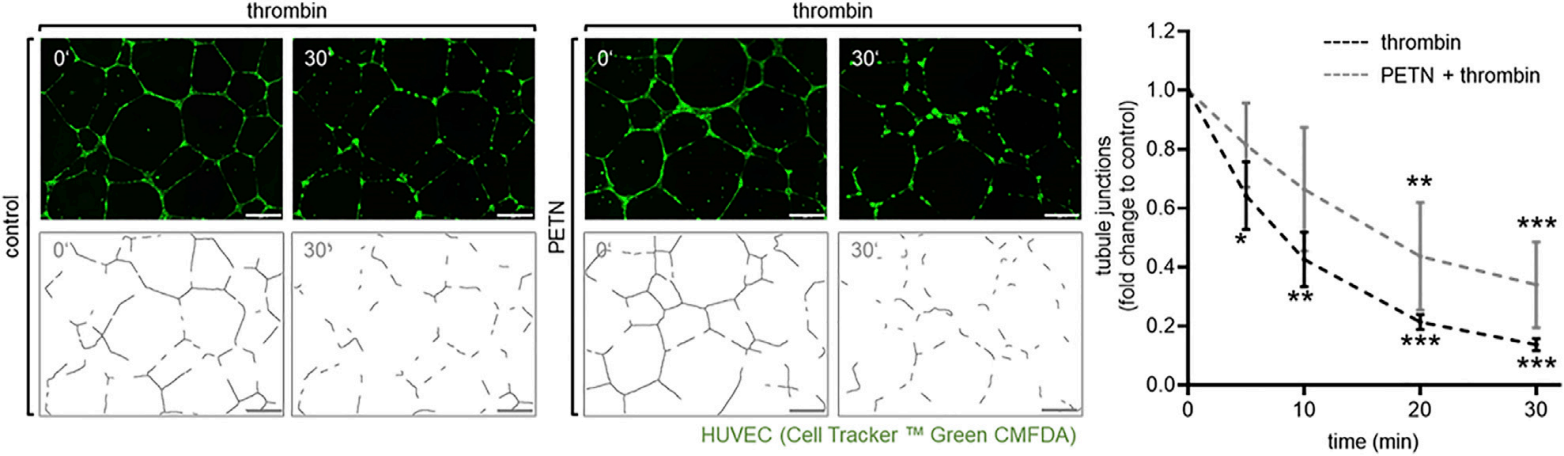
Treatment with PETN leads to a delay in thrombin-induced disruption of tubular junctions. In an angiogenesis assay endothelial cells (visualised by CellTracker™ Green CMFDA) embedded in Matrigel™ formed tubes. Treatment with 10 U/ml thrombin for 5–30 min leads to a significant reduction of tubule junctions (dotted line, *p = 0.038, **p = 0.001, ***p < 0.001, compared to 0 min). After pretreatment with 50 µM PETN for 24 h the thrombin-induced disruption of tubular junctions is delayed and reduced (solid line, **p = 0.002, ***p < 0.001 compared to 0 min), (*n* = 3, representative picture, picture for evaluation, scale bar = 200 μm, and evaluation of numbers of junctions, mean value ±SEM, two-way RM ANOVA followed by Holm-Sidak’s multiple comparisons test).

In addition, we could show that the tubule junctions that were formed in Matrigel™ by HUVEC were significantly destroyed after the addition of 10 U/ml thrombin for 5–30 min (*p* < 0.05). However, pretreatment with 50 µM PETN led to a milder and delayed disruption of those junctions (Figure 4).

**FIGURE 5:**
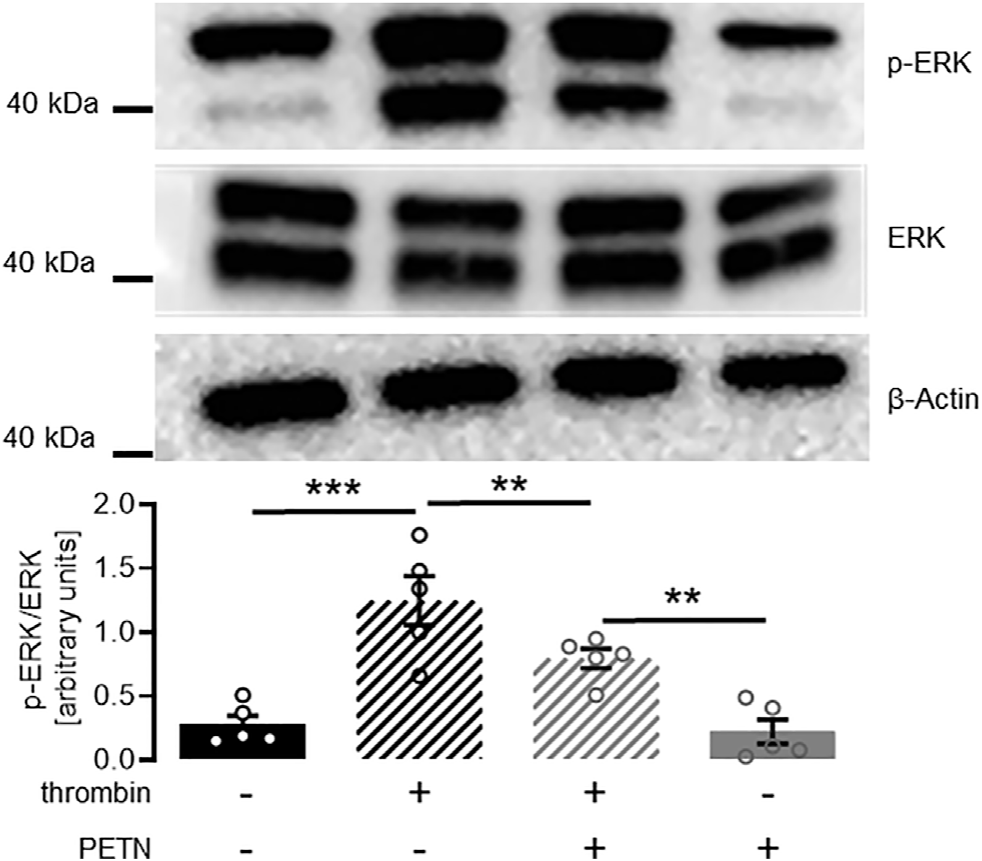
PETN treatment significantly reduces thrombin-induced ERK signaling. Stimulation of endothelial cells with 10 U/ml thrombin for 2 min leads to a profound ERK phosphorylation (black hatched bar, ***p < 0.001). Pretreatment with 50 µM PETN for 24 h does not induce basal ERK phosphorylation (grey bar). Thrombin treatment of PETN-pretreated cells still induces significant ERK phosphorylation (grey hatched bar, **p = 0.003). The thrombin-effect, however, is significantly reduced by 37 ± 14% compared to cells that were not pretreated with PETN (**p = 0.004). (*n* = 5, representative blot and densitometry, mean value ±SEM, two-way RM ANOVA followed by Holm-Sidak’s multiple comparisons test).

Having observed a protective effect of PETN on endothelial cell function, we wanted to investigate the mechanism behind this. In a western blot experiment we could show that treatment of HUVEC with 10 U/ml thrombin for 2 min led to a profound and significant ERK-phosphorylation (*p* < 0.001). In presence of 50 µM PETN the amount of phosphorylated ERK significantly decreased by 37 ± 14%. (*p* = 0.004, Figure 5).

**FIGURE 6:**
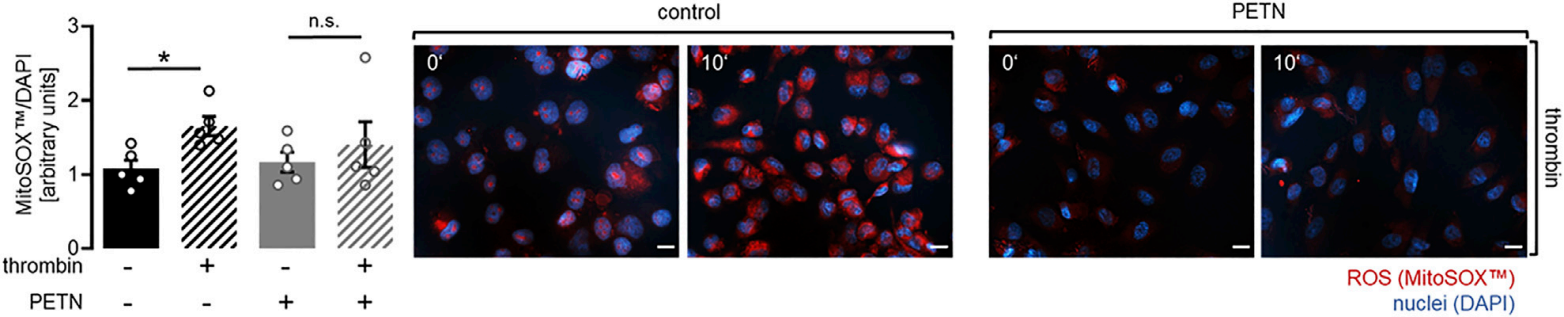
PETN treatment inhibits thrombin-induced ROS production. Treatment of endothelial cells with 10 U/ml thrombin for 10 min leads to a significant 63 ± 29% increase in ROS compared to control as detected in a staining using the MitoSOX™ reagent (black hatched bar, *p = 0.046). Pretreatment with 50 µM PETN for 24 h reduces thrombin-induced ROS production (grey hatched bar) (*n* = 5, representative picture, scale bar = 20 μm, and quantification, mean value ±SEM, two-way RM ANOVA followed by Holm-Sidak’s multiple comparisons test).

Upregulation of HO-1 and SOD2 in HUVEC pretreated with 50 µM PETN led to the assumption that PETN-mediated protective effects on endothelial cells were the result of the reduction of ROS in endothelial cells pretreated with PETN. To investigate if ROS was produced in HUVEC in response to thrombin treatment we used the MitoSOX™ reagent. Staining and evaluation showed that treatment of HUVEC with 10 U/ml thrombin for 10 min led to a significant 63 ± 29% increase in ROS production compared to control cells (*p* = 0.046, Figure 6). MitoSOX™ staining of HUVEC pretreated with 50 µM PETN for 24 h showed that the effect of thrombin on ROS production was significantly reduced and there was only a 21 ± 20% increase in ROS production (Figure 6).

## Discussion

This project focuses on the effect of PETN on functional changes within HUVEC induced by a high concentration of thrombin that represent endothelial dysfunction like breakdown of barrier, impairment of angiogenesis and induction of stress pathways. Our study demonstrates that treatment of HUVEC with 50 µM PETN for 24 h led to a significant upregulation of HO-1 but did not influence the expression of eNOS. 10 U/ml thrombin led to a stress response in endothelial cells as seen by increased ROS production, ERK phosphorylation and a disruption of barrier function and angiogenesis. These stress effects were significantly attenuated by pretreatment of HUVEC with PETN. PETN inhibited the effects of thrombin and therefore protected the endothelial cells on a molecular level. We propose that PETN protects endothelial cells from dysfunctional changes like disruption of angiogenesis and barrier breakdown by reducing stress-induced ROS production, presumably *via* the upregulation of HO-1 and the subsequent inhibition of NADPH-Oxidase.

Literature shows that PETN treatment of endothelial cells leads to activation of the antioxidant protein HO-1 (Dragoni et al., 2007; Schuhmacher et al., 2010). HO-1 is the rate limiting enzyme which catalyzes the degradation of heme to CO and bilirubin (Sikorski et al., 2004; Hull et al., 2014; Daiber and Munzel, 2015). The antioxidative effect of HO-1 is potentiated by the antioxidative properties of bilirubin. Indeed our data confirm that PETN significantly increases HO-1 mRNA levels under basal conditions. In addition, the increase in mRNA expression of SOD2 further underlines the antioxidant properties of PETN. Furthermore PETN is reported to restore eNOS activity (Schuhmacher et al., 2010). We could not observe changes in mRNA expression levels of eNOS upon treatment with PETN. However, eNOS activity was not evaluated in our experimental settings.

This hypothesis is further supported by our findings that there is no reduction in basal ROS levels after treatment of HUVEC with PETN. This coincides with our finding that HUVEC treated with PETN did form a stable barrier comparable to untreated cells and also formed a tubular network in Matrigel™, which did not differ from the network formed by untreated cells. Taken together these data show that PETN does not induce abnormal cell behavior under basal conditions and thus, does not lead to excessive and potentially harmful overstabilization of the endothelial barrier or excessive angiogenesis.

Since our project focuses on the protective effect of PETN under preeclamptic conditions we looked for a suitable stimulus to mimic endothelial dysfunction as seen during PE. One hallmark of this clinical syndrome is organ malperfusion, which stems from the systemic increase in endothelial permeability. A factor known to increase endothelial permeability is thrombin. Literature data shows that there is an excessive thrombin generation in the placenta during PE (Lockwood et al., 2007; Huang et al., 2015) which makes thrombin a suitable model. Nevertheless, keeping in mind, that *in vivo* the induction of endothelial dysfunction is more complex and not only dependent on one factor. Our data depicts that treatment of HUVEC with thrombin at the high concentration of 10 U/ml, indeed leads to a stress response, which can be seen by disturbed angiogenesis, barrier breakdown, ERK phosphorylation and ROS production.

In literature several pathways are proposed by which thrombin induces barrier disruption. On the one hand it is reported that thrombin, *via* the activation of its receptor protease-activated receptor 1 (PAR1), leads to an increase in intracellular Ca^2+^ and a subsequent activation of the Myosin light chain kinase (MLCK). MLCK phosphorylates MLC, which leads to contraction of the cell and thus an increase in permeability. Another proposed mechanism is that thrombin, *via* protease activated receptor one and subsequent NADPH-Oxidase activation, leads to ROS production which induces ERK phosphorylation (Bogatcheva et al., 2002; Coughlin, 2005; Andrikopoulos et al., 2015). ERK phosphorylation induces MLC phosphorylation by Rho-mediated inhibition of the MLC2 phosphatase (Wei et al., 2011; Aslam et al., 2012). Phosphorylated ERK itself contributes to barrier dysfunction by phosphorylating junction proteins which leads to a disassembly of tight junctions (Makarenko et al., 2014).

In our experiments using the xCELLigence^®^ system we could show that thrombin treatment of a stable endothelial barrier led to a fast increase of permeability as shown by a reduced cell index. Pretreatment of HUVEC with PETN could slow down, but not completely prevent, this process. We did not find any literature data showing evidence that PETN can protect from an increase in intracellular Ca^2+^ concentration and a subsequent MLCK activation. However, we could show that thrombin treatment of HUVEC leads to a strong ERK phosphorylation, as well as an increased ROS production. Both effects were significantly reduced after pretreatment with PETN. Nevertheless, the barrier disrupting pathways involving ERK and ROS only make up a part of the thrombin effect on endothelial barrier. This could explain why PETN only partially protects from endothelial barrier breakdown after thrombin stimulation. These results lead to the assumption that the protective effect of PETN on endothelial cells is primarily of an antioxidative nature.

A disturbance in correct angiogenesis *in vivo* is one of the causes of placental malperfusion during PE and FGR. Thrombin is known to support and strengthen angiogenesis (Blackburn and Brinckerhoff, 2008; Andrikopoulos et al., 2015). At the same time, at higher concentrations this effect is reversed and thrombin leads to apoptosis and disturbed angiogenesis (Catar et al., 2021). In our experiments treatment of a network of tubules formed by HUVEC on Matrigel™ with thrombin led to a disruption of tubular junctions. When cells were pretreated with PETN this effect was significantly delayed. Literature shows that PETN enhances endothelial function through antiproliferative and antiapoptotic mechanisms (Oppermann et al., 2009). We propose that this is mediated *via* a protection from oxidative stress and ROS-induced cell damage.

One of the main enzymes responsible for ROS production is NADPH oxidase which is dormant under resting conditions, but can be activated under stress, for example by high concentrations of thrombin (Huang et al., 2015). It is known that HO-1 leads to inhibition of NADPH oxidase activity which results in reduced ROS formation (Schuhmacher et al., 2010). A proposed mechanism is that bilirubin inhibits the assembly and therefore activation of NADPH oxidase (Datla et al., 2007; Luo et al., 2019). In our experiments thrombin, which was used to mimic preeclamptic conditions, led to a significant increase in ROS. Endothelial cells pretreated with PETN showed significantly lower thrombin-induced ROS levels which demonstrates the antioxidative effect of PETN *via* HO-1 under stress conditions. Hence we propose, that PETN protects endothelial cells on a molecular level mainly through its antioxidative properties by inhibiting NADPH oxidase and consequently preventing ROS production and ROS-induced cell damage.

In contrast to our experimental setup there are a variety of factors that lead to endothelial dysfunction and increased permeability during PE. Oxidative stress probably being the main inducer of the endothelium damaging effects seen in PE and FGR.

**FIGURE 7:**
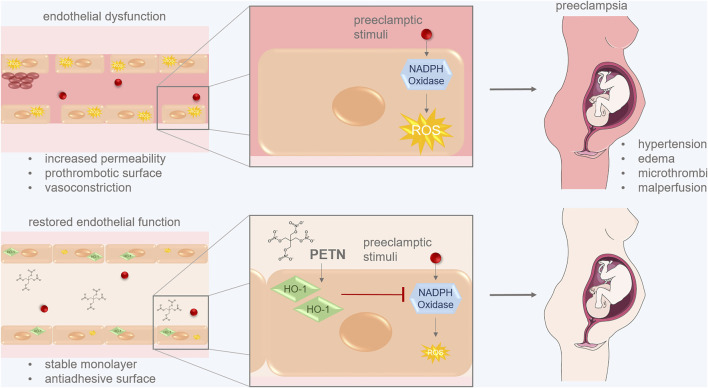
PETN can protect endothelial cells from pregnancy-induced dysfunction. Preeclamptic stimuli lead to increased ROS production *via* the activation of NADPH oxidase. This induces endothelial dysfunctions leading to symptoms of preeclampsia like hypertension, edema, microthrombi and malperfusion. PETN treatment of endothelial cells induces expression of the antioxidative HO-1 which leads to reduced ROS by inhibiting NADPH oxidase activity. This restores endothelial function which ensures a stable monolayer and an antiadhesive surface of vessels. These changes reduce symptoms of PE. (picture of pregnant woman from SMART:smart.servier.com).

To summarize we revealed that PETN can protect endothelial cells from pregnancy-induced dysfunction and through stabilizing endothelial resistance and angiogenic properties. PETN most likely acts *via* the upregulation of HO-1 and the subsequent reduction of stress-induced ROS and effects of oxidative stress (Figure 7). This confirms our hypothesis that PETN-induced improvement in endothelial health takes place on a molecular level and consequently contributes to less severe symptoms of PE and FGR.

## Data Availability

The authors confirm that the data supporting the findings of this study are available within the article. Raw data are available from the corresponding author, upon reasonable request.

## Abbreviations

ECGM, endothelial cell growth medium; eNOS, endothelial NO Synthase; ERK, extracellular signal-regulated kinase; FGR, fetal growth restriction; HO-1, heme oxygenase 1; HUVEC, human umbilical vein endothelial cells; MLC, myosin light chain; MLCK, myosin light chain kinase; NO, nitric oxide; PE, preeclampsia; PETN, Pentaerytrithyltetranitrate; ROS, reactive oxygen species; SOD2, superoxide dismutase 2.
